# HFE Gene Mutations in Cryptogenic Cirrhosis Patients

**Published:** 2012-01-20

**Authors:** Hossein Sendi, Marjan Mehrab-Mohseni

**Affiliations:** 1 The Liver-Biliary-Pancreatic Center, Cannon Research Center, Carolinas Medical Center, Charlotte, NC, USA; 2 Department of Biology, University of North Carolina at Charlotte, Charlotte, NC, USA

**Keywords:** Hemochromatosis, Iran, C282y, H63D

Dear Editor,

In Western countries, HFE-linked hereditary hemochromatosis (HH) is considered to be the most common cause of iron overload. The HFE gene, first identified in 1996, is located on the short arm of chromosome 6. The majority of patients with phenotypic HH are homozygous for the C282Y mutation, a major mutation of the HFE gene, whereas compound heterozygosity (C282Y/H63D) is found in patients with a milder iron loading phenotype [1]. Because hepatic iron overload promotes liver fibrogenesis in HH, it is crucial to know whether the presence of any HFE gene mutations might accelerate liver damage.

In a study by Jowkar et al. [2] published in the November issue of Hepatitis Monthly, the C282Y mutation was not detected in cryptogenic cirrhosis patients as well as healthy individuals; however, 22% of the patients and 28% of the healthy individuals were found to be heterozygous for the H63D mutation. Therefore, they concluded that HH is not the major cause of cryptogenic cirrhosis in the Iranian population. Although this study adds to the general knowledge on the molecular genetics of HH in the different regions of Iran, the findings of this study should be debated. We, as well as other researchers, have studied the frequency of HFE gene mutation among Iranian patients with chronic hepatitis and healthy individuals [3][4][5]. In the aforementioned studies, it was found that the allele frequency of the C282Y mutation was between 0% and 0.1%, whereas the allele frequency of the H63D mutation varied between 8.6 and 12.5% in healthy individuals. It is also noteworthy to mention that cryptogenic cirrhosis is a diagnosis of exclusion. Therefore, it is assumed that several of the patients in these studies are in fact alcoholic hepatitis or nonalcoholic steatohepatitis (NASH) patients. The patients' reluctance to admit to alcohol consumption along with the absence of advanced molecular techniques result in a higher number of false positive cases of cryptogenic cirrhosis in Iran. It is also not quite plausible to conclude that "HH is not a major cause of cryptogenic cirrhosis in the Iranian population" as Jowkar et al. have indicated in their study [2]. Perhaps, the authors meant to say that the development of cirrhosis in these patients cannot be attributed to HFE mutations. In the absence of any positive laboratory index of iron overload, there would be no evidence to correlate merely HFE mutations with hepatic cirrhosis even if these patients harbored HFE mutations. Taken together, our understanding of the role of HFE mutations in liver fibrogenesis in the context of viral hepatitis, alcoholic hepatitis, or other unknown accelerating factors is very premature, and further research and broader translational studies are required to unravel the underlying mechanisms.
